# Estimating body mass and composition from proximal femur dimensions using dual energy x-ray absorptiometry

**DOI:** 10.1007/s12520-018-0665-z

**Published:** 2018-06-18

**Authors:** Emma Pomeroy, Veena Mushrif-Tripathy, Bharati Kulkarni, Sanjay Kinra, Jay T. Stock, Tim J. Cole, Meghan K. Shirley, Jonathan C. K. Wells

**Affiliations:** 1 School of Natural Sciences and Psychology, Liverpool John Moores University, Byrom Street, Liverpool, L3 3AF UK; 2 Deccan College Postgraduate and Research Institute, Pune, India; 3 National Institute of Nutrition, Hyderabad, India; 4 Department of Non-communicable Disease Epidemiology, London School of Hygiene and Tropical Medicine, London, UK; 5 ADaPt Project, PAVE Research Group, Department of Archaeology and Anthropology, University of Cambridge, Cambridge, UK; 6 UCL Great Ormond Street Institute of Child Health, UCL, London, UK

**Keywords:** Lean mass estimation, Fat mass estimation, India, Archaeology, Forensics, DXA

## Abstract

Body mass prediction from the skeleton most commonly employs femoral head diameter (FHD). However, theoretical predictions and empirical data suggest the relationship between mass and FHD is strongest in young adults, that bone dimensions reflect lean mass better than body or fat mass and that other femoral measurements may be superior. Here, we generate prediction equations for body mass and its components using femoral head, neck and proximal shaft diameters and body composition data derived from dual-energy x-ray absorptiometry (DXA) scans of young adults (*n* = 155, 77 females and 78 males, mean age 22.7 ± 1.3 years) from the Andhra Pradesh Children and Parents Study, Hyderabad, India. Sex-specific regression of log-transformed data on femoral measurements predicted lean mass with smaller standard errors of estimate (SEEs) than body mass (12–14% and 16–17% respectively), while none of the femoral measurements were significant predictors of fat mass. Subtrochanteric mediolateral shaft diameter gave lower SEEs for lean mass in both sexes and for body mass in males than FHD, while FHD was a better predictor of body mass in women. Our results provide further evidence that lean mass is more closely related to proximal femur dimensions than body or fat mass and that proximal shaft diameter is a better predictor than FHD of lean but not always body mass. The mechanisms underlying these relationships have implications for selecting the most appropriate measurement and reference sample for estimating body or lean mass, which also depend on the question under investigation.

## Introduction

Research continues to address the problem of estimating body mass from the skeleton since body size is an important characteristic of a species or population linking many aspects of their behaviour, diet, mortality risk and life history (Charnov 1993; Harvey and Clutton-Brock 1985; Harvey and Read 1988; Robson and Wood 2008; Sibly and Brown 2007; Will et al. 2017). Secular trends in body size (height and mass) in recent centuries are also of significant interest for the insight they offer into temporal changes in living conditions and their implications for contemporary growth and health, particularly in relation to obesity-linked conditions (Ng et al. 2014; Xi et al. 2012). It is important to adjust for body mass when examining evolutionary changes in the relative size of organs such as the brain (e.g. McHenry 1988; Ruff et al. 1997) or to standardise bone properties to infer activity levels using limb bone cross-sectional geometry (Ruff 2008), which require body mass to be estimated. Body mass is also an important characteristic in forensic profiling (Houck 2017; Moore and Schaefer 2011).

A number of studies have focussed on using femoral head diameter (FHD) to estimate body mass, and the three most commonly used equations are those of McHenry (1992), Grine et al. (1995) and Ruff et al. (1991). Femoral head size is assumed to relate to loading (body mass) at the end of growth before the femoral head fuses, after which no changes in the size of the joint occur regardless of changes in loading due to mass or activity (Lieberman et al. 2001; Ruff et al. 1991; Trinkaus et al. 1994). The same principle is thought to apply to other joint surfaces, so joint dimensions of other major long bones and the first metatarsal have also been used to estimate body mass (Chevalier et al. 2018; De Groote and Humphrey 2011; Elliott et al. 2016a, b; Grabowski et al. 2015; Grine et al. 1995; Lorkiewicz-Muszyńska et al. 2013; McHenry 1992; Moore 2008; Moore and Schaefer 2011; Ruff 2007; Ruff et al. 2018; Squyres and Ruff 2015; Wheatley 2005; Will and Stock 2015).

An alternative to such ‘mechanical’ approaches is Ruff’s morphometric method (Ruff 1991) which uses stature and bi-iliac breadth to estimate body mass. This method requires good skeletal preservation and despite compound estimation errors when applied to skeletons (estimating stature and living bi-iliac breadth and in turn body mass), it offers somewhat better reliability than predictions based on joint sizes (Ruff et al. 1991, 2005; Schaffer 2016).

Many of the FHD equations have relatively high associated errors (e.g. Ruff et al. 1991 report standard errors of estimate (SEE) of ≥ 14%), and they have been found to be unreliable when the equations are applied to individuals of known body mass (Chevalier et al. 2016; Elliott et al. 2016a; Heyes and MacDonald 2015). For example, Elliott et al. (2016a) report that in a cadaveric sample of European origin, estimated body mass using FHD was only within 20% of true body mass for 58% of females and 76% of males using the best-performing equations. This may in part be because studies such as Elliott et al.’s (2016a, b) examined older individuals from wealthier countries, where weight gain in middle and later adulthood can be considerable and obesity is an increasing problem. Such trends may weaken the relationship between joint size (reflecting mass in early adulthood) and body mass. Squyres and Ruff (2015) analysed distal femoral dimensions of young adults for this reason and recorded reduced SEEs of 9.9–13.2% for body mass estimation. In contrast, Elliott et al. (2016b) did not find consistently improved results when the equations they derived from a variety of postcranial measurements were based only on individuals aged 18–39 years compared with the full range (to 91 years). (See Ruff et al. 2018 for a further review of previous studies and their limitations).

Some evidence suggests that joint sizes and other external bone dimensions may be most strongly related to skeletal muscle or lean mass (Baker et al. 2013; Chumlea et al. 2002; Himes and Bouchard 1985; LeBrasseur et al. 2012; Pomeroy et al. 2018; Reeves 2014; Semanick et al. 2005; Taes et al. 2009; Wu et al. 2007) and only weakly to fat mass (Bailey and Brooke-Wavell 2010; Beck et al. 2001, 2009; Cole et al. 2012; El Hage and Baddoura 2012; Farr et al. 2014; Hu et al. 2012; Leslie et al. 2014; Mallinson et al. 2013; Moon et al. 2015; Pomeroy et al. 2018; Semanick et al. 2005; Sioen et al. 2016; Taes et al. 2009; Travison et al. 2008; Wu et al. 2007) and thus show weaker relationships to total body mass. This closer relationship of bone dimensions to lean mass than to fat mass may result from the functional relationship between bone and skeletal muscle (Edwards et al. 2013; Fricke and Schoenau 2007; Judex et al. 2016; Parfitt 1997; Puthucheary et al. 2015; Rauch and Schoenau 2001; Schoenau 2005; Schoenau and Fricke 2006; but see, e.g. Judex et al. 2016) and/or shared developmental origins (DiGirolamo et al. 2013; Karasik et al. 2009; Lang et al. 2009; Mikkola et al. 2009; Seeman et al. 1996). A closer relationship between bone properties and lean mass than fat mass would mean that bone dimensions give particularly poor body mass estimates for recent, relatively adipose samples. It may also be that other bone measurements, such as femoral neck or shaft diameter (Elliott et al. 2016b; Pomeroy et al. 2018; Ruff et al. 1991), are more sensitive to actual mechanical loads and thus may prove better predictors of body mass and its components.

The potential to estimate different components of body mass from the skeleton is of interest since humans are characterised by relatively high body fat and low skeletal muscle mass (a major constituent of lean mass) compared with other primates (Muchlinski et al. 2012; Zihlman and Bolter 2015) and fossil hominins such as Neanderthals (Churchill 1998, 2006; Trinkaus 1983; Trinkaus et al. 1991; Wells 2010, 2017). Furthermore, different human populations are known to vary widely in body composition. For example, South Asians have relatively low lean mass in proportion to height and total body mass, which is implicated in their elevated susceptibility to type 2 diabetes (reviewed in Wells et al. 2016), while Pacific Islanders have high lean mass relative to height and total body mass, which is hypothesised to reflect cold stress experienced while at sea (Houghton 1996; Wells 2012; Wilberfoss 2012). The ability to estimate lean and fat mass from the skeleton would therefore enable us to investigate when and why such inter- and intra-specific differences in body composition arose.

The aim of this study is to derive new equations for body, lean and fat mass estimation using measurements of the proximal femur derived from dual-energy x-ray absorptiometry (DXA) scans of living young adults of known body mass and estimated body composition. We test the hypotheses that (1) lean mass can be more reliably estimated from skeletal measurements than fat mass or total body mass and (2) other bone measurements (femoral neck and shaft dimensions) are equally good, if not superior, for predicting body mass and its components than FHD.

## Materials and methods

Whole-body and regional hip DXA scans of young adult participants in the Andhra Pradesh Children and Parents Study (APCAPS) were used in this study. APCAPS is a large, intergenerational epidemiological study of children, their parents and siblings living in villages surrounding Hyderabad, India (see Kinra et al. 2014 for an overview). The study was approved by the ethics committees of the National Institute of Nutrition, Hyderabad, and the London School of Hygiene and Tropical Medicine, and participants provided informed consent.

Sample selection has been described previously (Pomeroy et al. in press), but briefly, participants underwent whole-body and regional hip and lumbar spine DXA scans at various stages of APCAPS, and scans from the third survey wave (2010–2012) were selected to ensure that as many participants as possible were in their early 20s, and so had completed their growth: the participants selected were aged between 20 and 26 years. A random stratified sample containing equal numbers of males and females was selected to give even coverage across the range of height and body mass. Only the whole-body and regional hip scans were used in our analyses.

All DXA scans were performed on a Hologic Discovery A (Bedford, MA, USA) at the National Institute of Nutrition, Hyderabad, India. The scanner was calibrated daily during the study, and the left hip scanned for bone density analysis. Stature was measured using a Leicester Height Measure (Chasmors, Camden, London, UK) to the nearest centimetre, and body composition was estimated from whole-body DXA scans taken at the same time using inbuilt software (version 12.5). Standard software options were used to calculate the total lean mass and fat mass. Weight was measured to the nearest 0.1 kg in light clothes without footwear using a digital Seca scale (www.seca.com).

**Fig. 1:**
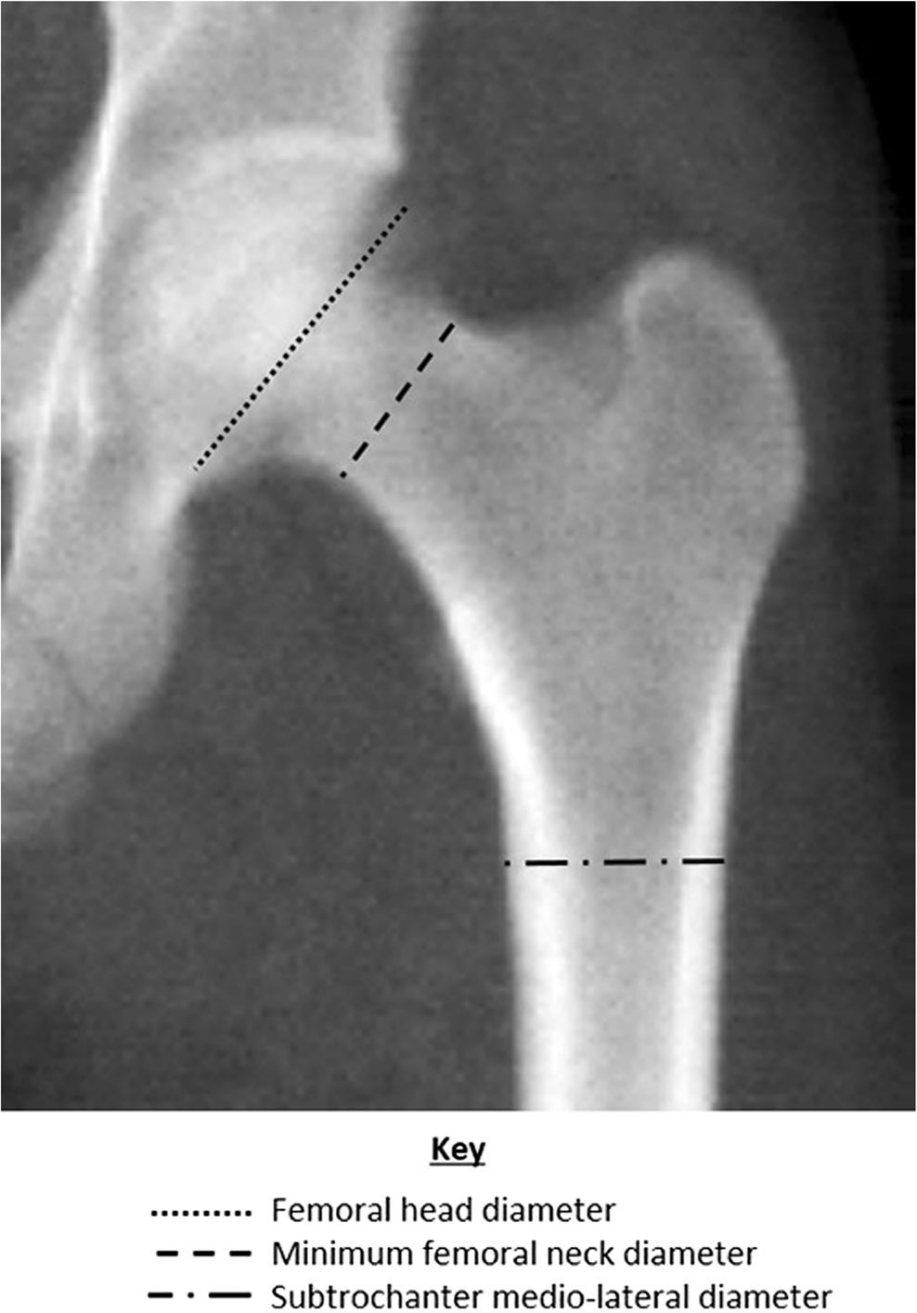
Example of a hip DXA scan showing measurements collected in this study

The proximal femur scan ‘P’ files were exported from the Hologic APEX software and opened in ImageJ (Rasband 1997–2016) using the P Reader plugin developed by Minxuan Dong (Dr Neil Dong, pers. comm. 2015). The automatic brightness and contrast adjustment in ImageJ was applied to enhance the clarity of the image in a standard manner, and images were scaled using known hip scan area dimensions provided by the manufacturer. Supero-inferior head diameter, minimum diameter of the femoral neck, and mediolateral diameter of the subtrochanteric region of the femur were measured using the line measurement tool in ImageJ following osteological definitions of these measurements (Bräuer 1988; Martin and Saller 1957) as closely as possible (Fig. 1). All measurements were taken by two of the authors (EP and VM) and the mean of their measurements used in subsequent analyses.

The repeatability of the DXA measurements (intra- and inter-observer error) were assessed using the technical error of measurement (TEM) and the coefficient of reliability (*R*), calculated following Ulijaszek and Lourie (1994). We also calculated TEM as a percentage of the mean for that measurement (%TEM). Inter-observer error was calculated from all data collected by EP and VM, while intra-observer error was calculated from repeated measurements of 10 individuals taken by EP at least 1 day apart. While there are no universally accepted objective limits for TEM, %TEM or *R*, the results in Table 1 indicate that intra-observer error is low: The coefficient of reliability was ≥ 0.93 and TEM ≤ 2.4%. This compares with *R* ≥ 0.86 and TEM ≤ 3.3% for inter-observer error, suggesting that the repeatability of the measurements is fairly good. An additional limitation is that the resolution of the scans, which for this dataset is approximately 2 pixels per millimetre, may also affect measurement accuracy even when measurement to the sub-pixel level is enabled in ImageJ. However, the averaging of multiple measurements will help to improve the reliability of the measurements.

**Table 1:** Intra- and inter-observer reliability statistics for proximal femoral measurements derived from left hip DXA scans

Measurement	Intra-observer			Inter-observer		
	TEM (mm)	%TEM	*R*	TEM (mm)	%TEM	*R*
Supero-inferior head diameter	0.76	1.8	0.96	1.4	3.3	0.86
Supero-inferior neck diameter	0.64	2.4	0.93	0.7	2.7	0.93
Subtrochanteric mediolateral shaft diameter	0.29	1.0	0.99	0.7	2.3	0.91

*TEM* technical error of measurement, *R* coefficient of reliability

### Processing DXA measurements

The Hologic Discovery A uses a fan beam of x-rays which leads to magnification in the mediolateral plane of the body, but not the cranio-caudal plane (Boudousq et al. 2005; Griffiths et al. 1997). The extent of this effect depends on the distance of the object of interest (in this case, the proximal femur) from the source. Bone dimensions that are not orientated cranio-caudally on the DXA scans therefore need to be corrected for underlying tissue thickness.

The relationship between degree of magnification and distance from the scanner bed was assessed by scanning a stepped calibration block supplied by the manufacturer. This has three metal plates measuring 100 mm square set on an acrylic block at 40, 95 and 210 mm above the base. This block was scanned in full-body mode in three positions on the bed (in the midline oriented longitudinally on the bed, and oriented mediolaterally with the higher end to the right side and then to the left). The metal plates were measured using Mitutoyo sliding callipers to the nearest 0.1 mm, and also on each of the scans using ImageJ. The percentage magnification of each plate measurement was calculated with reference to the calliper measurements of the same plates. The percentage magnification was then plotted against the known height of each plate above the base of the acrylic block. An ordinary least squares (OLS) regression line was fitted to these data, and the equation to estimate percentage magnification based on height above the table was derived (Eq. 1):

1 $$ \mathrm{Magnification}\ \left(\%\right)=100-\left(-{0.186}^{\ast }\ \mathrm{height}\ \mathrm{above}\ \mathrm{table}\ \mathrm{in}\ \mathrm{millimetres}+131.5\right) $$

The Hologic Discovery A DXA images of the hip include an estimate of total tissue thickness at the hip based on x-ray attenuation (*T*), equivalent to inches of acrylic. To transform this to body tissue equivalent, *T* must be multiplied by 1.18 to account for differences in density of these materials (T.L. Kelly, Hologic Inc., pers. comm. August 2015), and converted to centimetres by multiplying by 2.54.

2 $$ \mathrm{Total}\ \mathrm{body}\ \mathrm{thickness}\ \left(\mathrm{cm}\right)=1.18\times T\times 2.54 $$

This gives a total body thickness, but the height of the proximal femur above the bed must be estimated to correct the actual bone measurements. McKay et al. (2005) state that based on computed tomography observations, the femur lies in the mid-sagittal plane around the hip, but they do not present the data on which this is based. Pocock et al. (1997) demonstrated that among 30 Australian women, aged 32–65 years, the mean height of the femoral head above the scanning bed was 11.2 cm (range 7.1–15.8 cm). The Hologic software gives total tissue thickness at the hip, but it is unclear what total body thickness was in the Australian dataset and so whether our dataset is comparable.

To test the height of the femur at the hip in a supine position, we measured the height of the femoral head above the bed in pelvic MRI scans of 53 young women of South Asian heritage living in London, UK, collected by MKS as part of a separate research project. MRI scans were 3D volumetric T_2_-weighted acquisitions (144 contiguous coronal slices; TR 15.5 ms; TE 5.1 ms, flip angle 25°, voxel size 1.2 mm^3^) performed at Great Ormond Street Hospital for Children NHS Trust using a 3T Siemens Magnetom Prisma scanner (Siemens, Erlangen, Germany). The height of the centre of the femoral head from the bed was measured in OsiriX version 8.5 (Rosset et al. 2004). Mean height of the centre of the femoral head above the table was 61% of body thickness (standard deviation 2.5%). The degree of magnification for a given individual was then calculated using Eq. 1, where

3 $$ \mathrm{Height}\ \mathrm{above}\ \mathrm{table}\ \left(\mathrm{cm}\right)=0.61\times \mathrm{total}\ \mathrm{body}\ \mathrm{thickness} $$

To correct bone measurements for magnification of the hip scans in the mediolateral, but not cranio-caudal direction, the linear measurement (for example FHD) and the angle of the measurement as reported in ImageJ were taken, and adjusted measurements were calculated using Pythagoras’ theorem on an imaginary right angle triangle constructed treating the ImageJ length as the hypotenuse. These new adjusted measurements were then used in subsequent analyses.

Ordinary least squares (OLS) regression was used to derive equations to estimate body, lean and fat mass from each of the proximal femur measurements. While there is ongoing discussion regarding the most appropriate regression model to use for such analyses (Elliott et al. 2016b; Ruff et al. 2012; Smith 2009), others have reported that reduced major axis (RMA) regression produces greater average errors (Elliott et al. 2016b) and it is less appropriate for multivariable regression.

Regression models were calculated for each of the individual femoral measurements for males and females separately, given established sex differences in body composition. Natural logarithms of all data were used to account for potential allometry and non-normality in the distribution of some variables (Glazier 2013; Huxley 1932; Sokal and Rohlf 1987). Models were also calculated for the raw data but yielded slightly higher SEEs (data not shown), so log-transformed data were preferred. Given variability in archaeological preservation, equations based on single measurements are potentially most useful, but a model containing multiple measurements might offer greater accuracy where all measurements can be taken. We therefore also ran a forward stepwise multiple regression model including all three femoral measurements as potential predictors. The relative performance of the regression models was assessed from the adjusted *R*^2^ values and SEEs. All analyses were conducted using SPSS for Windows v. 24.0 (IBM Corporation, Chicago), with *p* values < 0.05 considered significant.

## Results

**Fig. 2:**
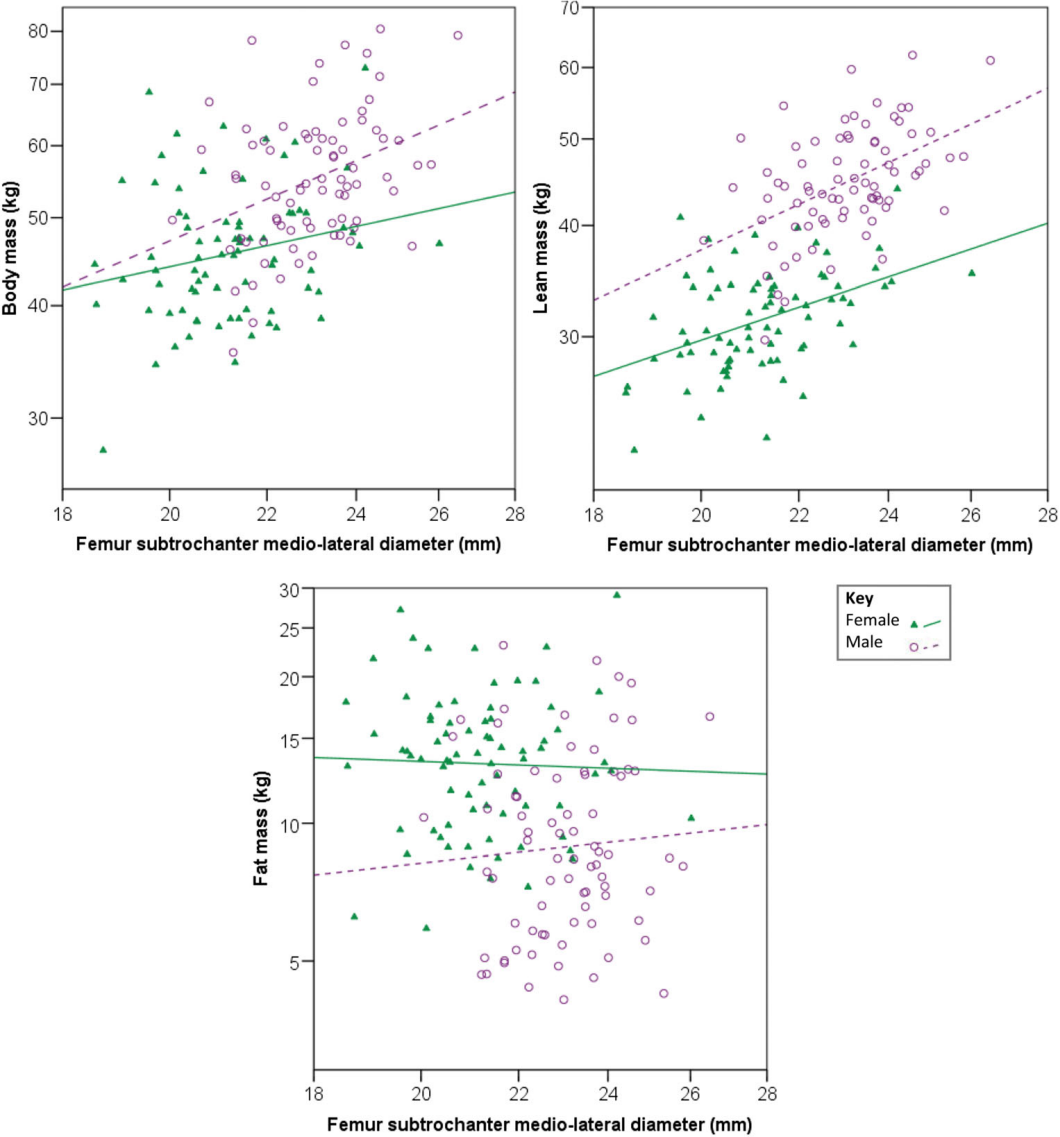
Scatterplots of body, lean and fat mass against femur subtrochanter mediolateral diameter. Axes are drawn on a natural log scale

The characteristics of the study sample are summarised in Table 2. Mean age was 22.7 years, with even numbers of males and females. The regression models are presented in Tables 3 and 4 for females and males, respectively. For both sexes, lean mass could be estimated most reliably from measurements of the proximal femur (SEE = 12.0–13.5%), while the estimation of body mass was less reliable (SEE = 15.9–16.9%) and fat mass prediction showed poor reliability with statistically non-significant models (SEE = 33.5–44.5%). It should be noted that for females, the regressions for body mass were also non-significant for femoral neck and subtrochanteric shaft diameter. Example scatterplots for body, lean and fat mass against femur subtrochanter mediolateral diameter are shown in Fig. 2; those for other femoral measurements are similar (not shown). For lean mass in females and both body and lean mass in males, the subtrochanteric mediolateral diameter yielded better models with lower SEEs and adjusted *R*^2^ values, while for females, FHD was the best predictor of body mass.

**Table 2:** Demographic characteristics of the study sample

Variable	Combined sex (*n* = 155)	Females (*n* = 77)	Males (*n* = 78)
Age (years)	22.7 (1.3: 20.3–25.6)	23.0 (1.3: 20.4–25.6)	22.4 (1.2: 20.3–24.9)
Height (cm)	160.3 (9.2: 138.0–180.0)	153.8 (6.4: 138.0–166.0)	166.8 (6.6: 147.0–180.0)
Body mass (kg)	51.1 (10.1: 27.6–80.5)	46.2 (8.1: 27.6–72.9)	55.9 (9.6: 35.5–80.5)
Body mass index (kg/m^2^)	19.7 (2.9: 13.7–28.8)	19.5 (3.0: 13.7–28.8)	20.0 (2.9: 15.4–28.7)
Lean mass (kg)	38.3 (8.6: 22.2–61.9)	31.5 (4.2: 22.2–43.9)	45.1 (6.3: 29.7–61.9)
Fat mass (kg)	11.8 (5.0: 4.0–29.0)	13.9 (4.6: 5.9–29.0)	9.7 (4.4: 4.0–23.0)
Femur head diameter (mm)	38.9 (3.2: 31.4–50.8)	36.8 (2.2: 31.4–42.0)	41.1 (2.5: 36.4–50.8)
Femur neck diameter (mm)	24.5 (2.4: 19.3–30.4)	22.8 (1.6: 19.3–26.3)	26.1 (1.8: 21.9–30.4)
Femur subtrochanter shaft diameter (mm)	22.1 (1.6: 18.5–26.4)	21.2 (1.4: 18.5–26.0)	23.0 (1.2: 20.0–26.4)

Values given as mean (standard deviation: range)

**Table 3:** Regression equations for estimating body, lean and fat mass from measurements of the proximal femur in females

Equation	*r*	Adjusted *R*^2^	%SEE	*p* value
Body mass				
1.377 + 0. 678 × head	0.24	0.05	16.5	0.03
2.732 + 0.348 × neck	0.15	0.02	16.9	0.2
2.100 + 0.563 × subtrochanter	0.22	0.04	16.7	0.06
Lean mass				
0.536 + 0.854 × head	0.37	0.12	12.5	0.001
1.573 + 0.599 × neck	0.32	0.09	12.7	0.004
0.697 + 0.899 × subtrochanter	0.44	0.19	12.0	< 0.001
Fat mass				
1.276 + 2.287 × head	0.07	− 0.01	33.5	0.6
2.681–0.031 × neck	0.01	0.00	33.5	0.9
3.104–0.170 × subtrochanter	0.03	− 0.01	33.5	0.7

Note that all variables are natural logs. Raw bone diameters originally in millimetres, and raw mass in kilogrammes

%SEE = SEE × 100 as natural log transformation of the data results in SEEs which are already percentages when multiplied by 100 (Cole 2000; Cole and Altman 2017)

*Head* femoral head super-inferior diameter, *Neck* femoral neck minimum superior-inferior diameter, *Subtrochanter* femur subtrochanter mediolateral diameter

**Table 4:** Regression equations for estimating body, lean and fat mass from measurements of the proximal femur in males

Equation	*r*	Adjusted *R*^2^	%SEE	*p* value
Body mass				
0.876 + 0.844 × head	0.30	0.08	16.3	0.007
2.219 + 0.512 × neck	0.29	0.07	16.3	0.01
0.505 + 1.117 × subtrochanter	0.36	0.12	15.9	0.001
Lean mass				
0.649 + 0.848 × head	0.36	0.12	13.2	0.001
1.876 + 0.590 × neck	0.30	0.08	13.5	0.007
− 0.080 + 1.237 × subtrochanter	0.48	0.22	12.4	< 0.001
Fat mass				
− 0.105 + 0.615 × head	0.08	− 0.006	44.5	0.5
− 1.088 + 1.001 × neck	0.16	0.01	44.1	0.2
0.397 + 0.568 × subtrochanter	0.07	− 0.008	44.6	0.5

Note that all variables are natural logged. Bone diameters are in millimetres, and mass in kilogrammes

%SEE = SEE × 100 as natural log transformation of the data results in SEEs which can be viewed as percentages when multiplied by 100 (Cole 2000; Cole and Altman 2017)

*Head* femoral head super-inferior diameter, *Neck* femoral neck minimum superior-inferior diameter, *Subtrochanter* femur subtrochanter mediolateral diameter

Although the regression coefficients for lean mass on bone measurements were similar for males and females, in a pooled-sex analysis, sex was highly significant when added as a term to the model, but the interaction between sex and bone measurement was not (results not shown). This indicates that while the regression lines by sex are parallel, they are not coincident, so sex-specific estimation equations are preferable.

The stepwise regression procedure based on all three femoral variables resulted in only one statistically significant multivariable equation. This equation was to predict lean mass among women from FHD and mediolateral subtrochanteric femoral shaft diameter, although this only reduced the SEE by 0.2% compared with the best univariable model (data not shown).

## Discussion

Our study demonstrates that in a sample of young adults from the region around Hyderabad, India, lean mass can be estimated from measurements of the proximal femur with an SEE of 12.2%. The estimation of body mass is less reliable with SEEs around 16.5%, while fat mass is only poorly estimated (SEE ≥ 35.5%)—indeed, bone measurements were not significant predictors of fat mass. It is worth noting that there is no widely accepted or objectively defined standard for acceptable rates of error for such estimation equations. While statistics such as the proportion of individuals whose estimates were within 10, 15 or 20% of known values (e.g., Elliott et al. 2016a; Lorkiewicz-Muszyńska et al. 2013; Ruff et al. 2005), these are arbitrary thresholds. The performance of equations is best judged by taking into account errors associated with estimates generated for a target individual/sample and the purpose for which the estimates are being derived.

In terms of relative accuracy, the errors associated with our equations are similar to or smaller than those reported for equivalent equations from other samples, although thorough comparisons are hampered by the fact that different studies report different measures of error associated with their equations. We therefore focus our comparisons on studies reporting a comparable %SEE statistic. Ruff et al. (1991) reported SEEs for body mass estimation of 14.4% or greater based on FHD, neck diameter and estimated subtrochanter cross-sectional area for a single-sex and -ancestry sample from the USA (average age 53 years). Using distal femoral dimensions in a sample of young US adults, Squyres and Ruff (2015) reported SEEs of 11.5–12.2% for body mass, while Ruff (2000) reported SEEs of 6–8% for estimating body mass from bi-iliac breadth and stature, and Schaffer (2016) reported similar errors of 5–8% for sex- and ancestry-specific equations based on bi-iliac breadth and stature in the third US National Health And Nutrition Examination Survey dataset.

Our young adult sample gave mostly smaller associated errors than those reported by Ruff et al. (1991). With the exception of sex- and ancestry-specific equations for white females, which had %SEEs of 14.4% for body mass, SEEs for body mass in that study ranged from 16.5–24.1%. However, we note that there are differences between these samples other than the age of participants which might explain the difference in results. Our sample was of entirely South Asian ancestry from a restricted region around Hyderabad and from communities undergoing urbanisation and a transition from traditional to more westernised lifestyles. Thus BMI, body mass and stature, and variation in these characteristics, are likely to be relatively low in our sample compared with Ruff et al.’s pooled-sex and -ancestry US sample. Only mean body mass can be compared with Ruff et al. (1991), but this was 80.8 and 72.4 kg for males and females, respectively, in their sample, compared with 55.9 and 46.2 kg, respectively, in our sample. Although our results are broadly consistent with the proposal that using a young adult sample will give more accurate body mass prediction equations, we cannot demonstrate that this is the definitive explanation and the effects of more homogenous ancestry, lower BMI, lower body fat and smaller stature might also be responsible. We also note that the improvements represented by our equations were modest compared with using distal femur measurements from a young US adult sample (Squyres and Ruff 2015).

It is notable that equations for estimating lean mass had lower SEEs than those for body mass, while SEEs were high for equations to estimate fat mass. This is consistent with results of previous studies which suggest that limb bone dimensions and cross-sectional properties are more closely related to lean mass than to fat mass (Baker et al. 2013; Chevalier et al. 2018; Chumlea et al. 2002; Himes and Bouchard 1985; LeBrasseur et al. 2012; Pomeroy et al. 2018; Reeves 2014; Semanick et al. 2005; Taes et al. 2009; Wu et al. 2007).

The closer relationship between lean mass and bone properties may be because the greatest forces acting on bones come from muscle action rather than general body mass effects due to gravity (Baker et al. 2013; Beck et al. 2001; Burr 1997; Capozza et al. 2004; Hsu et al. 2006; Petit et al. 2005; Robling 2009; though see Ruff 2003), and/or due to shared developmental factors affecting muscle and bone (DiGirolamo et al. 2013; Karasik et al. 2009; Lang et al. 2009; Mikkola et al. 2009; Seeman et al. 1996). Our results, with those and other studies, imply that poorer correspondence between body mass and bone dimensions in older, westernised populations (Elliott et al. 2016b; Ruff et al. 1991) may be at least in part due to greater adiposity at all ages in such populations, rather than the disruption of a functional link between body mass at the end of growth and joint size by later weight gain. Although lean mass increases with increased body fat, the former does not keep pace with the latter in overweight or obese individuals (Forbes 1999; Wells and Victora 2005), and this may therefore weaken the relationship between body mass and bone properties. Studies testing existing equations (Young et al. 2018) or generating new ones (Chevalier et al. 2018) have reported improved reliability when analyses exclude individuals who are likely to have an unusually high or low proportion of body fat (i.e., those with BMI outside the normal range of 18.5 to 24.9 kg/m^2^). These results may indicate that the extremes of percentage body fat are not well reflected in femoral measurements due to a weak link between adiposity and skeletal properties.

For all measurement locations, subtrochanteric mediolateral shaft diameter provided the best-performing models. A note of caution is however necessary, as properties of the proximal femoral shaft relate to body breadth (Davies and Stock 2014; Weaver 2003), although this may be less of a problem for body mass estimation since body breadth is itself an important determinant of body mass (Ruff et al. 2005; Ruff 2000; Schaffer 2016). Potentially more problematic are age- and activity-related influences on long bone shaft morphology.

It is well accepted that shaft dimensions and cross-sectional geometry are related to activity levels (e.g., Haapasalo et al. 2000; Pearson and Lieberman 2004; Ruff et al. 2006; Ruff and Hayes 1983; Shaw and Stock 2009a, b; Stock and Pfeiffer 2001; Trinkaus et al. 1994) and extreme body mass (Agostini and Ross 2011; Reeves 2014). This contrasts with joint sizes which are thought to be fixed by the time the epiphysis fuses in adolescence and to show little relation to activity levels (Lieberman et al. 2001; Reeves 2014; Ruff 1988; Ruff et al. 1991; though see Eckstein et al. 2002). However, the period of greatest responsiveness of the shaft cross-sectional properties to mechanical loading (mass and activity) is also widely considered to be late adolescence and early adulthood (Bertram and Swartz 1991; Forwood and Burr 1993), and approximately 80% of the variation in cross-sectional geometry of human long bone shafts seems to be determined by body mass (Davies 2012). Thus, while changes in body mass and behaviour during adulthood may create some noise in the data, they may not invalidate the use of shaft cross-sectional properties to estimate body mass and its components. However, body mass estimation equations based on shaft cross-sectional properties may be problematic if applied to populations whose activity level differs widely from that of the reference population.

It is also known that long bone shafts undergo age-related expansion of the periosteal margin which could influence external diameters (Feik et al. 2000; Garn et al. 1967; Lazenby 1990a, b; Ruff and Hayes 1983). The use of shaft dimensions to estimate body or lean mass should therefore be cautious until the extent of age- and activity-related influences on shaft properties are more fully quantified and their relationship to early adulthood and current body or lean mass (or at time of death) are better understood.

Studies seeking to estimate body mass should consider the choice of measurements and reference samples carefully, and the purpose of estimating body or lean mass for a given study needs to be taken into account when selecting an appropriate estimation method (Pomeroy et al. 2018; Ruff et al. 2018). The lack of plasticity in femoral head dimensions render them less suitable for estimating body mass at the time of death, particularly in older individuals who may have accumulated greater excess body weight. However, there are circumstances where estimating body or lean mass before the variable, age-related accumulation of excess weight is actually of greater interest, such as in the study of adaptive evolutionary trends in body mass or composition, which may be more evident in young adulthood (e.g. Hruschka et al. 2014; see Pomeroy et al. in press for further discussion). The interpretation of what we might consider early adult ‘peak phenotype’, when selective pressures might be expected to be strongest and sexual dimorphism is greatest, is easier when less obscured by environment-specific ‘noise’ created by later mass accumulation.

Conversely, bone shaft properties may be more appropriate estimators where body mass at death is needed, such as in forensic cases (though the poor correspondence with fat mass here should still be noted). In such cases, the choice of reference sample is also likely to be more critical: equations may give inaccurate body or lean mass estimates when applied to study samples differing significantly in body composition and/or activity from the reference sample. It is important that sufficient detail concerning reference samples (including summary statistics on stature, body mass, BMI and body composition, where available) are provided when estimation methods are described so that the suitability of the reference sample for a given application can be readily assessed.

In order to apply our equations and convert estimates back to original units (i.e. mass in kg), the antilog of the calculated value should be used. While others have argued for a correction to counteract ‘detransformation bias’ (Smith 1993), this is unnecessary since it assumes that the OLS model based on the raw data is the ‘best’ model and adjusts the results of the log-log model to more closely reflect the results of the raw data regression. However, the log-log regression models the error in a different way to the raw data analysis that is not necessarily inferior.

The study has a number of strengths, including the fact that the sample was composed of young adults for whom body mass was measured (and not obtained through recall, as in Ruff et al. 1991) and composition was estimated using a consistent method. The range of statures and body mass are relatively wide, and the sample also derives from a population where marked obesity even in young adulthood is uncommon, though not completely absent as Table 2 shows. Given the greater proportion of body fat observed in South Asians, values around, for example, 28 kg/m^2^ represent a similar level of body fatness to a BMI of 30 kg/m^2^ or even greater among Europeans (Rush et al. 2009; Tillin et al. 2015; WHO Expert Consultation 2004). Nonetheless, the relatively low rate of obesity may make the equations more applicable to past populations where obesity is thought to have been less prevalent. This could, however, be a limitation to applying the methods in forensic cases.

Several additional caveats are warranted in interpreting the results and applying our equations. The sample was entirely of South Asian ancestry, and it is currently unknown whether the relationship between measurements of the proximal femur and body, lean and fat mass is consistent across populations of different genetic backgrounds. The results of Ruff et al. (1991) suggest that there may be some variation in this relationship. Therefore, our equations should be applied cautiously until it has been established whether any such interpopulation variation in the underlying relationship exists. The need for population-specific stature estimation equations is well documented and widely recognised (e.g. Auerbach and Ruff 2010; Holliday and Ruff 1997; Nat 1931; Pan 1924; Pomeroy and Stock 2012; Stevenson 1929).

The relatively short stature and low body mass of our sample compared with other populations worldwide should also be noted, since it is widely accepted that OLS regression equations cannot be applied to individuals falling outside the range of the original data (e.g. Smith 2009). According to the national level data from the NCD-RisC group, India’s 1996 birth cohort ranked 192nd and 178th out of 200 countries for mean female and male stature, respectively (NCD Risk Factor Collaboration 2016), and 192nd and 190th for female and male BMI, respectively, in 2016 (NCD Risk Factor Collaboration 2017). Even within South Asia, urban populations have higher average BMIs than rural populations (e.g. Ebrahim et al. 2010), so careful consideration must be given as to whether our reference sample is sufficiently similar when applying our equations to other populations.

Our analysis demonstrates that using DXA scans is a feasible method which could be used to investigate potential variation in the relationship between bone dimensions and body mass and its components among groups of different geographical origin or ancestry (see also Wheatley 2005). The considerable number of large-scale epidemiological studies of worldwide populations where DXA scans have been performed offer great potential in terms of suitable datasets.

In conclusion, our results show that lean mass can be predicted with smaller associated error than body mass, while fat mass cannot be reliably predicted, and that subtrochanteric mediolateral shaft diameter is a better predictor of lean mass in both sexes and body mass in males only than femoral head or neck diameter. The implication is that FHD may not scale to total load (body mass) as is often assumed, but that bone properties and lean mass are linked through either forces generated by muscle or by shared genetic or developmental factors between lean mass and bone. Nonetheless, as lean mass is the major component of body mass especially in younger adulthood, measurements such as FHD do provide a useful estimator of early adult or ‘ideal’ body mass. A better understanding of the link between lean mass and bone morphology presents the possibility for a more nuanced investigation of variation of body size in the past. Further testing of the way in which femoral dimensions covary with activity, body mass, age and ethnicity are needed to confirm the wider applicability of equations for estimating lean and body mass generated in this and other studies, and large-scale epidemiological studies involving DXA scans offer datasets with wide global coverage. The emerging complexity of the relationships between body mass or its components and bone properties suggests there would be value in broadly reconsidering new approaches to body mass estimation from the skeleton.
